# A survey of variable selection methods in two Chinese epidemiology journals

**DOI:** 10.1186/1471-2288-10-87

**Published:** 2010-09-29

**Authors:** Huimin Liao, Henry S Lynn

**Affiliations:** 1 Department of Biostatistics, School of Public Health, Key Laboratory on Public Health Safety of the Ministry of Education, Fudan University, Shanghai 200032, China

## Abstract

**Background:**

Although much has been written on developing better procedures for variable selection, there is little research on how it is practiced in actual studies. This review surveys the variable selection methods reported in two high-ranking Chinese epidemiology journals.

**Methods:**

Articles published in 2004, 2006, and 2008 in the Chinese Journal of Epidemiology and the Chinese Journal of Preventive Medicine were reviewed. Five categories of methods were identified whereby variables were selected using: A - bivariate analyses; B - multivariable analysis; e.g. stepwise or individual significance testing of model coefficients; C - first bivariate analyses, followed by multivariable analysis; D - bivariate analyses or multivariable analysis; and E - other criteria like prior knowledge or personal judgment.

**Results:**

Among the 287 articles that reported using variable selection methods, 6%, 26%, 30%, 21%, and 17% were in categories A through E, respectively. One hundred sixty-three studies selected variables using bivariate analyses, 80% (130/163) via multiple significance testing at the 5% alpha-level. Of the 219 multivariable analyses, 97 (44%) used stepwise procedures, 89 (41%) tested individual regression coefficients, but 33 (15%) did not mention how variables were selected. Sixty percent (58/97) of the stepwise routines also did not specify the algorithm and/or significance levels.

**Conclusions:**

The variable selection methods reported in the two journals were limited in variety, and details were often missing. Many studies still relied on problematic techniques like stepwise procedures and/or multiple testing of bivariate associations at the 0.05 alpha-level. These deficiencies should be rectified to safeguard the scientific validity of articles published in Chinese epidemiology journals.

## Background

Selecting the appropriate variables for an analytical model is an important task in epidemiological research. This may involve finding the right combination of confounders to adjust for when estimating the association between an exposure variable and the disease outcome, obtaining a parsimonious set of prognostic variables in the construction of a screening instrument or predictive tool, or simply determining independent predictors for a clinical outcome in order to guide future research hypotheses.

The approaches that have been used in variable selection are diverse and plentiful, including stepwise methods (forward selection and/or backward elimination), best subsets regression, shrinkage methods (e.g. ridge regression, lasso), bootstrap adjustments, change-of-estimates methods, and use of directed acyclic graphs and prior knowledge [1-13]. In particular, automated techniques have been especially popular in the past, perhaps because they are discussed in nearly all elementary textbooks on applied statistics and implemented in many commercial statistical software packages. Such techniques are however notorious for underestimated standard errors and inflated significance levels, inclusion of irrelevant variables and exclusion of authentic predictors, and unstable solutions even with minor changes in the data [2,4,8,13-17].

Although much research has been done on comparing existing variable selection procedures and proposing new solutions, little has been written on the type and quality of variable selection strategies found in existing journals. One prior study provided an assessment of general statistical analyses in five Chinese medical journals [18], but this investigation is the first to document the variable selection methods that are reported in Chinese epidemiology journals.

## Methods

All original articles published in 2004, 2006, and 2008 in the Chinese Journal of Epidemiology and the Chinese Journal of Preventive Medicine were reviewed. These two journals were ranked 1^st ^and 2^nd^, respectively, according to the 2004 List of Core Chinese Public Health Journals. We searched for articles using electronic retrieval based on the keywords "variable selection", "variable screening", and "multivariable", and hand searched all articles that were not selected electronically in order not to miss any articles that may involve selection of variables. The variable selection methods identified in the articles were then classified into five mutually exclusive categories: Category A - methods that selected variables based only on bivariate associations; Category B - methods that selected variables based only on their performance in a multivariable regression model; Category C - methods that first screened variables based on their bivariate associations, and then selected those screened-in variables according to their performance in a multivariable regression model; Category D - methods that selected variables based on their bivariate associations or their performance in a multivariable regression model (i.e. the methods in categories A and B were both used to select variables); Category E - methods that selected variables using other criteria; e.g. prior knowledge or personal judgment, and tree models.

## Results

Of the 1882 original articles published in the two journals, 287 (15%) described how variables were selected in their data analyses. There was a greater proportion of articles in the Chinese Journal of Epidemiology (231/1199 = 19%) that reported using variable selection methods compared with the Chinese Journal of Preventive Medicine (56/683 = 8%), but there were no substantial differences in the proportion of articles using variable selection methods between 2004, 2006, and 2008. The majority of the 287 studies were cross-sectional designs (51%), followed by case-control (30%) and cohort (14%) designs. Logistic regression models were most prevalent (76%), followed by linear regression models (11%) and Cox regression models (6%). Most of the studies considered around 10 to 30 potential variables for selection, although one study examined a maximum of 105 variables.

As shown in Table 1, nearly a third (category C, 30%) of the 287 articles screened variables first according to the statistical significance of bivariate associations (e.g. correlations, chi-square statistics), and then performed further selection via a multivariable regression model using either stepwise algorithms (45%) or significance testing of the individual regression coefficients (33%). Twenty-two percent, however, did not specify the mechanism used for selecting the final variables in the multivariable model. The second most commonly used strategy (category B; 26%) was variable selection via a multivariable model. Again, stepwise algorithms (46%) and significance testing of individual regression coefficients (45%) were the preferred methods, but 9% of the articles also did not specify how they selected the final variables. Category D, selection of variables using either bivariate associations or multivariable models, made up 21% of the articles. Slightly more studies here employed significance testing of individual regression coefficients (47%) compared to stepwise algorithms (42%), but 11% did not provide information on how the variables were selected in the multivariable model. The least utilized variable selection method was significance testing based solely on bivariate associations (category A, 6%), and 83% (15/18) of these studies designated significance with a 0.05 alpha-level. The remainder of the studies (category E, 17%) selected variables primarily according to prior knowledge or personal judgment (90%, 45/50).

**Table 1:** Characteristics of Variable Selection Methods in 287 Articles

Category	Frequency	Percent
A: Select via bivariate associations	18	6
B: Select via multivariable model	74	26
^†^*Stepwise*	34	46
^†^*Significance testing of individual regression coefficients*	33	45
^†^*Unspecified*	7	9
C: Screen by bivariate associations then select via multivariable model	85	30
^†^*Stepwise*	38	45
^†^*Significance testing of individual regression coefficients*	28	33
^†^*Unspecified*	19	22
D: Select via bivariate associations or multivariable model	60	21
^†^*Stepwise*	25	42
^†^*Significance testing of individual regression coefficients*	28	47
^†^*Unspecified*	7	11
E: Select using other criteria	50	17
*Prior knowledge or personal judgment*	45	90
*Tree model*	4	5
*Factor analysis followed by stepwise regression*	1	2

^†^Variable selection method employed in multivariable model

Eighteen percent (51/287) of the studies were concerned with selecting confounders for adjustment. The three methods found among these studies were categories E (45/51, 88%), B (4/51, 8%), and A (2/51, 4%). In comparison, among the other 236 studies, the top three methods were categories C (85/236, 36%), B (70/236, 30%), and D (60/236, 25%).

The most common significance level chosen for selecting variables was 0.05. For example, 77% (65/85) and 83% (50/60) of the studies in categories C and D, respectively, selected variables using bivariate association tests at the 5% significance level, and 97% of the 89 studies in categories B, C, and D that selected variables based on significance tests of the individual regression coefficients in the multivariable model also used a 5% alpha-level. For the 97 studies in categories B, C, and D that used stepwise routines to select variables in their multivariable models, the forward/backward stepwise algorithm was most popular (37%, 36/97) followed by the backward elimination algorithm (16%, 16/97). It is disconcerting, however, that 60% (58/97) did not provide clear information on the specific algorithm used in the stepwise method and/or the significance levels used to select or remove variables in the algorithm.

## Discussion

Deciding independent predictors for a clinical outcome or selecting the appropriate covariates to serve as control variables are mainstays in epidemiological research, and their proper choice of techniques is essential in safeguarding correct conclusions. In the past decades, heightened attention has been devoted to developing sound methods for variable selection, but there is a lack of research on how variable selection methods are used in existing publications. Research efforts in public health in China have increased considerably since the 1980s, and currently there are over 200 Chinese journals addressing different areas within this field. This study provides a survey of the variable selection methods found in two top-tier Chinese epidemiology journals.

Although there is a multitude of procedures available for selecting variables, our survey indicates a paucity of methods actually reported in the Chinese Journal of Epidemiology and the Chinese Journal of Preventive Medicine. The most common method (category C) is screening of variables based on the statistical significance of bivariate associations, followed by variable selection in a multivariable model using either stepwise algorithms or significance testing of the individual regression coefficients. The popularity of screening variables for multivariable analysis was also observed in a review of manuscripts published from 1989 to 1994, covering journals such as the Annals of Internal Medicine, New England Journal of Medicine, Journal of the American Medical Association, Cancer, Circulation, and the American Journal of Public Health [19]. However, the screening of variables using significance testing runs the risk of increased type I errors of the predictors in the multivariable model [20], and should instead be based on evaluation of background knowledge [6]. In addition, using bivariate associations to select variables for multivariable analysis ignores potential confounding or collinearity between the independent variables, implying that a nonsignificant variable in the bivariate analysis can in fact be a significant variable in the multivariable analysis [19,21]. This shortcoming applies whether one is using bivariate analyses to screen variables or to select variables directly (category A). A fifth of the reviewed manuscripts selected variables either via bivariate analyses or multivariable analysis (category D). Such procedures create a further conundrum of how to reconcile the results from the two analyses, speculating why some selected factors should adjust for other variables while other factors need not.

Among the studies employing multivariable analyses, stepwise procedures were used most often even though the hazards of these automated variable selection methods have been attested for many years [8,14,16]. The 5% alpha-level was the preference for significance testing in the bivariate analyses, while a 5% alpha-entry-level and a 10% alpha-removal-level were the norm for studies that used stepwise routines. These significance levels, however, have been found to be too high for minimizing type I error [2], and also too low for minimizing type II error and attaining good predictive models [1,3,21,22]. More disturbingly, 60% of the studies that used stepwise procedures failed to specify the algorithm and/or the significance levels, and 15% (33/219) of the studies that utilized multivariable models did not mention how the variables were selected. The latter figure was very close to the 14% reported in a random sample of articles published in Lancet and the New England Journal of Medicine during the late 1980s [23]. Less than 6% of the studies that involved modeling included model diagnostics and underwent model validation. Such disappointing phenomenon was also apparent in a recent review of 99 articles in the Journal of Clinical Epidemiology and the American Journal of Epidemiology [24]. The use of prior knowledge or personal judgment was the basis for deciding how to select variables in 16% (45/287) of the manuscripts, with the majority of them concerned with choosing confounders. This is a positive sign although many of the explanations lacked substantive background information and references, and none provided causal inferential tools like directed acyclic graphs.

The two journals that were reviewed are by no means a random selection, although their high rankings may lead one to suspect that the problem represents just a tip of the iceberg among epidemiology journals in China. It would be interesting to investigate whether such phenomenon also holds true in other developing countries, especially those in Asia. The reasons behind the methodological deficiencies in the surveyed manuscripts are multi-faceted, but lack of training and loose regulation are perhaps the foremost culprits. Topics like shrinkage methods, bootstrap adjustments, and direct acyclic graphs are not part of the standard syllabus for preventive medicine and epidemiology students, who may only have two or three biostatistics courses. The proliferation of journals and submitted manuscripts in recent years has made it more difficult for editors to recruit qualified reviewers and enforce strict methodological requirements. It is also true that old habits die hard [17], and some researchers may continue to use deficient methods simply because they are easy to implement and journals do not seem to mind. To address these problems, journals can regularly publish editorials that focus on methodology in order to educate authors, enlist more biostatisticians to be on the editorial and review board, and instate manuscript guidelines on statistical analyses. More statistical training for our public health students is obviously desirable, but given the difficulty of squeezing extra courses into already packed curriculums it may be more pragmatic to foster greater collaboration between biostatisticians and public health scientists and let each person excel in their specialty.

## Conclusions

In summary, this assessment brings to light the deficiencies in how many studies in preventive medicine and epidemiology conduct variable selection, over-relying on multiple significance testing and stepwise regression algorithms. Nonetheless, these barriers are not insurmountable given coordinated and collaborative efforts by public health scientists and epidemiologists both within China and abroad. Better alternatives to variable selection; e.g. shrinkage methods [6,7], and use of direct acyclic graphs and subject matter knowledge [11,12], should be encouraged. If automated variable selection methods have to be used, then they should undergo bootstrap adjustments [5,8,9]. Neglect, on the other hand, should not be an option as continual ignorance may seriously undermine the scientific validity of epidemiological research in China.

## Competing interests

The authors declare that they have no competing interests.

## Authors' contributions

All authors made substantial contribution to the manuscript. HL participated in the design of this research, performed the literature review and analyses, and drafted the manuscript. HSL planned the design of this study, and participated in the methodological content and writeup of the manuscript. All authors have read and approved the final manuscript.

## Pre-publication history

The pre-publication history for this paper can be accessed here:

http://www.biomedcentral.com/1471-2288/10/87/prepub
